# A postpartum enriched environment rescues impaired cognition and oxidative markers in aged mice with gestational inflammation

**DOI:** 10.1002/brb3.2817

**Published:** 2022-11-21

**Authors:** Yu‐Xin Zhang, Qi‐Yao Wei, Ya‐Tao Wang, Li‐Ping Zeng, Shi‐Yu Sun, Yong‐Fang Wu, Chong‐Yang Ren, Fang Wang, Gui‐Hai Chen, Lei Cao

**Affiliations:** ^1^ Department of Neurology (Sleep Disorders) the Affiliated Chaohu Hospital of Anhui Medical University Hefei (Chaohu) Anhui P. R. China; ^2^ Department of Neurology the Second Affiliated Hospital of Anhui Medical University Hefei Anhui Province P. R. China; ^3^ Department of Neurology Bengbu Second People's Hospital Bengbu Anhui Province P. R. China; ^4^ Department of Neurology The First Affiliated Hospital of Anhui University of Science and Technology Huainan Anhui Province P. R. China; ^5^ Department of Neurology the First Affiliated Hospital of Anhui Medical University Hefei Anhui Province P. R. China

**Keywords:** aging, enriched environment, lipopolysaccharide, memory, S‐nitrosoglutathione reductase (GSNOR), tet methylcytosine dioxygenase 1 (TET1)

## Abstract

**Introduction:**

Previous studies have shown that gestational inflammation can accelerate age‐associated cognitive decline (AACD) in maternal mice; enriched environments (EEs) have been reported to protect normally aging mice from AACD and improve mitochondrial function. However, it is unclear whether the nitrosative stress‐related proteins tet methylcytosine dioxygenase 1 (TET1) and S‐nitrosoglutathione reductase (GSNOR) are involved in the accelerated aging process of gestational inflammation and whether EEs can slow this process.

**Methods:**

In this study, CD‐1 female mice on the 15th day of pregnancy were injected with bacterial lipopolysaccharide (50 μg/kg; LPS group) or an equivalent amount of normal saline (CON group) from the abdominal cavity for 4 consecutive days. Twenty‐one days after delivery, half of the LPS‐treated mice were randomly selected for EE until the end of the behavioral experiment (LPS‐E group). When the female rats were raised to 6 months and 18 months of age, the Morris water maze (MWM) was used to detect spatial learning and memory ability; RT‐PCR and Western blots were used to measure the mRNA and protein levels of hippocampal TET1 and GSNOR.

**Results:**

As for the control group, compared with 6‐month‐old mice, the spatial learning and memory ability of 18‐month‐old mice decreased, and the hippocampal TET1 and GSNOR mRNA and protein levels were decreased. Gestational inflammation exacerbated these age‐related changes, but an EE alleviated the effects. Pearson's correlation analysis indicated that performance during the learning and memory periods in the MWM correlated with the levels of hippocampal TET1 and GSNOR.

**Conclusions:**

Our findings suggest that gestational inflammation accelerates age‐related learning and memory impairments and that postpartum EE exposure could alleviate these changes. These effects may be related to hippocampal TET1 and GSNOR expression.

## Graphical abstract

Protein cysteine residues can be oxidized by nitric oxide (NO) to form protein S‐nitrosothiols (SNOs) in a process called S‐nitrosylation. S‐nitrosoglutathione reductase (GSNOR) is a highly specific denitrase for GSNO; it controls the intracellular levels of GSNO and protein SNOs and protects the body from nitrosative stress.

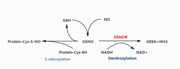

## 1 INTRODUCTION

Age‐associated cognitive decline (AACD) is a common symptom of aging (Wadhwa et al., 2018). Brain aging is a complex multi‐factor process. Negative life events such as material deprivation, bacterial infections, and low education levels are key risk factors, (Weiner et al., 2015), while positive life events have a protective effect on brain aging. Among them, normal reproductive experience or maternal experience can alleviate AACD (Li et al., 2016). However, due to significant changes in the endocrine and immune systems of the body, pregnancy also increases the sensitivity to adverse factors and especially increases the risk of infection (Oskvig et al., 2012). Lipopolysaccharide (LPS) is a powerful endotoxin that is commonly used to simulate bacterial infection in experimental animals. Our previous studies indicated that maternal exposure to LPS during pregnancy accelerated AACD in the middle‐aged mother (Li, Wang et al., 2016; Sun et al., 2020). However, how gestational inflammation affects AACD in mothers in young and old age has not been elucidated.

The hippocampus plays an important role in normal learning and memory consolidation and AACD, because the structural and functional changes induced by aging in occur earlier than other brain regions (Mendelsohn Larrick, 2012; Belblidia et al., 2018). Studies have shown that prenatal stress in the rat causes long‐term spatial memory deficits and hippocampal abnormalities as detected by magnetic resonance imaging (Stamler et al., 2001). We previously also found that embryonic exposure to LPS‐induced inflammation led to age‐related spatial learning and memory impairment and corresponding hippocampal neurobiochemical changes (Chen et al., 2011; Li, Wang et al., 2016; Wu et al., 2020).

Cognitive function depends on neurotransmitter systems such as nitric oxide (NO) signaling, which exerts effects on cognition by means of S‐nitrosylation. Nitrosation reactions can change protein activity, localization, stability, and protein–protein interactions as well as modulate signaling and stress responses (Khan et al., 2021; Zhang et al., 2017). At the physiological level, NO generally facilitates neuronal differentiation, development, and survival. However, excessive NO can result in aberrant S‐nitrosylation of proteins and increase the incidence of senescence‐related diseases (Nakamura et al., 2013). Accumulating evidence indicates that mitochondrial dysfunction can contribute to the pathophysiology of age‐related diseases. Mitochondria are critical for neuronal energy metabolism, calcium regulation, myelination, and neuronal and glial apoptosis (Princz et al., 2018; Zhuang et al., 2020). Therefore, mitochondrial decay may be central to neuronal damage and cognitive decline (Cheng et al., 2018; Lai et al., 2017). Mitochondria have a large number of protein cysteine residues. These residues can be oxidized by nitric oxide (NO) to form S‐nitrosothiols (SNOs), named S‐nitrosylation. Excessive S‐nitrosylation of related proteins will directly affect mitochondrial dynamic activity, reduce the efficiency of aerobic metabolism, inhibit mitochondrial autophagy, and destroy mitochondrial homeostasis (Rizza & Cardaci et al., 2018a). We previously found correlations between mitochondrial quality control and AACD (Zhuang et al., 2020). S‐nitrosoglutathione reductase (GSNOR) is a key enzyme that catalyzes denitrosylation to balance S‐nitrosylation and protects the body from nitrosative stress (Beigi et al., 2012; Liu et al., 2001a). Studies have shown that whole‐brain GSNOR expression declines with age and that its deficiency can impair mitochondrial function due to nitrosative stress (Rizza & Cardaci et al., 2018a).

*Gsnor* expression is epigenetically controlled by cytosine methylation of CpG islands located in the promoter region (Rizza & Cardaci et al., 2018a). The demethylating activity of tet methylcytosine dioxygenase 1 (TET1), a member of the 2‐oxoglutarate‐dependent dioxygenases, is required to counteract gene silencing due to cytosine methylation, and, in turn, sustain *Gsnor* transcription(Rizza & Filomeni, 2018) . Therefore, TET1 is closely related to cell senescence by its regulation of *Gsnor* expression. However, whether TET1 and GSNOR are involved in the accelerated AACD of gestational inflammation remains unknown.

Enriched environment (EE) usually refers to a larger group living in a larger living space, with toys that change frequently, such as running wheels, experimental mouse tunnels, poplar toys, rings, and so on, and is a classic model used in neuroscience (Nithianantharajah & Hannan, 2006). Increasing evidence suggests that EEs can promote hippocampal neurogenesis in mice and induce mitochondrial activity, and these results are closely related to the improvement of impaired cognitive function (Zhuang et al., 2020). Studies have indicated that an EE can alleviate the age‐related cognitive impairment and deficits in learning and memory capabilities that result from gestational exposure to LPS (Sun et al., 2020).

We hypothesize that TET1 and GSNOR are involved in the accelerated aging process of LPS exposure in late pregnancy and the positive effect of EEs. In this study, we first identified whether gestational inflammation affected learning and memory in young (6‐month‐old) and aged (18‐month‐old) CD‐1 mice. Subsequently, we evaluated whether the protein and mRNA levels of TET1 and GSNOR were altered in the hippocampus of CD‐1 mice based on age and treatment. Finally, we determined the correlations between learning and memory abilities and the measured neurobiological indicators in the different age and treatment groups.

## 2 MATERIALS AND METHODS

### 2.1 Animals and treatments

**FIGURE 1:**
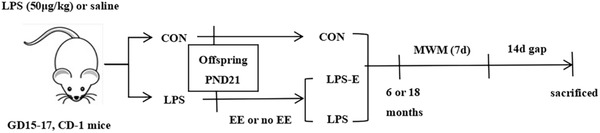
Timeline of experimental events. The pregnant mice were injected with LPS or saline from the abdominal cavity for four consecutive days starting from the 15th day of pregnancy. Weaned on the 21st day after childbirth, the mothers were divided into three groups based on whether the mice were exposed to an EE until the end of the experiment. The MWM test was performed at 6 and 18 months of age. Two weeks after the completion of the MWM test, the mice were sacrificed for follow‐up biochemical experiments. EE, enriched environment; CON, control group; LPS, lipopolysaccharide treatment group; LPS‐E, lipopolysaccharide plus enriched environment treatment group; MWM, Morris water maze

CD‐1 mice, 2 months old, 40 male mice, and 80 female mice, were purchased from the Model Animal Research Institute of Nanjing University. They were reared under standard experimental conditions with a 12‐hour light‐dark cycle, suitable temperature and humidity, and free eating and drinking. The model and breeding were based on our previous studies (Li, Cao et al., 2016; Wu et al., 2019). CD‐1 mice were caged at 1:2 (male: female) at 9:00 p.m. every night until the vaginal embolus was found and immediately separated. If there is no pregnancy in more than two weeks, the sample should be replaced. On the 15th day of pregnancy (28–34 g weight) were injected with bacterial lipopolysaccharide (50 μg/kg; LPS group) or an equivalent amount of normal saline (CON group) from the abdominal cavity for four consecutive days. On postnatal day 21, the mothers were separated from their offspring (this avoided the premature separation of mother and child, which would cause psychological stress to the mother and offspring) and breastfeeding was stopped. After the mother and child were separated, half of the mother mice that received LPS intraperitoneal injections were randomly selected for EE until the end of the behavioral experiment; these mice constituted the LPS‐E group. The mice of the CON group and LPS group were housed in standard plastic mouse cages (25.5 × 15 × 14 cm^3^, 4 mice per cage), and the mice of the LPS‐E group were housed in enlarged cages (52 × 40 × 20 cm^3^, 10−15 mice per cage). 6‐month‐old (6 M; young) and 18‐month‐old (18 M; aged) CD‐1 mice were used to complete the tests described in the sections below (10 per group in Morris Water Maze, six per group in the protein and mRNA markers measured). Mice with obvious defects were eliminated before the experiment. All procedures were approved by the Experimental Animal Ethics Committee of Anhui Medical University. The experimental process is shown in Figure 1.

### 2.2 Enriched environment

Right after breastfeeding was stopped, the LPS‐E mice were housed in a larger cage (52×40×20 cm^3^) to enrich social life in the group. Toys and novelty objects such as running wheels, experimental mouse mazes, swings, and ladders, were placed in the cage and constantly changed until the end of the behavioral experiment. This was done to expand the living space, provide places for exercise and escape as well as to provide stimulation to novel things, and enhance typical species behaviors of mice such as playing, fighting, and group sleep.

### 2.3 Morris water maze

Put a movable cylindrical escape platform with a diameter of 10 cm and a height of 24 cm into a black circular pool with a diameter of 150 cm and a height of 30 cm. Fill the pool with tap water at a suitable temperature and control the water level to be 1 cm higher than the platform. Surround the pool with a white curtain 50 cm away from the pool to shield the interference of external factors from the experiment, and paste 3 black markers of different shapes at equal distances on the curtain 1.5 m from the bottom of the pool as a space positioning clues for mice. The experiment process is divided into a learning (positioning and navigation) period and a memory (spatial exploration) period. The behavioral parameters (distance swam in the learning period, percent distance swam spent swimming in the memory period) were recorded and analyzed by behavioral analysis software (ANY‐Maze, USA). Learning period: During seven consecutive days of positioning navigation, the escape platform was 1 cm below the water surface and fixed in the center of the target quadrant. Only before the start of the experiment on the first day, the mice were placed on the platform for 30 s to adapt, and the mice facing the outer edge of the pool were randomly thrown into the water from any quadrant every day and were allowed to swim for a maximum of 60 s to find the platform. Each experimental mouse was trained four times a day, 15 min apart. Record the average swimming distance before finding the platform each time. The shorter the distance, the stronger the learning ability. Memory period: On the 7th day of the learning period, after completing four training sessions, the escape platform was taken out. After the mice recovered their strength, they entered the water again from the opposite quadrant of the platform quadrant (target quadrant) and swam arbitrarily in the pool for 60 s. The greater the percentage of the swimming distance in the target quadrant within 60 s of the total swimming distance, the stronger the memory ability.

### 2.4 Tissue preparation

In order to avoid the effects of the MWM test in the expression of Tet1 and Gsnor mRNA and protein in mouse hippocampus. We chose two weeks as a recovery period to reflect the more realistic results of the molecular experiments. Two weeks after the completion of the behavioral task, the mice were euthanized by cervical dislocation, but some mice whose health status is seriously affected in the MWM test could be excluded. The brain was removed by craniotomy and the hippocampus was then extracted and stored in an ultra‐low temperature refrigerator at −80°C. The right hippocampus was used for Western blotting (WB) and the left hippocampus was used for reverse transcription‐polymerase chain reaction (RT‐PCR).

### 2.5 Quantitative real‐time RT‐PCR (RT‐QPCR)

The hippocampus was mixed with TRIzol reagent following the manufacturer's instructions to obtain RNA. cDNA was extracted from RNA (1 μg) using The RevertAidTM First‐Strand cDNA Synthesis Kit. Took out cDNA as a template for quantitative real‐time PCR and performed amplification in a 10‐μL reaction mixture containing 5 uL of 2×SYBR Green mixture, 1 uL of each primer (10 uM), 1 uL of cDNA template, and 2 uL of RNase‐free water. The quantitative real‐time PCR reaction condition included one cycle of 95°Cfor 1 min and 40 cycles of 95°C for 20 s and 60°C for 1 min. The mRNA level was quantified using the 2^−ΔΔCt^ method. Beta‐actin served as the internal reference. The primer sequences are listed in Table 1.

**TABLE 1:** Sequences of the primers used for quantitative real‐time PCR

Target gene	Forward primer (5−3)	Reverse primer (5−3)
β‐actin	AGTGTGACGTTGACATCCGT	TGCTAGGAGCCAGAGCAGTA
GSNOR	TGGTTCTACTTGTGCCGTCT	AACGAGGACTTCCTGGATGG
TET1	GCTGTCTGATCCTTCTCCGA	CAAAGACATCCGGCTGTGAG

### 2.6 Western blotting

Tissue was lysed in protein‐neutral lysate, extract protein, and protein concentrations were determined using the bicinchoninic acid assay kit. After dissolving the protein to an equal concentration, add 5×SDS‐PAGE protein loading buffer according to 1:4 and heat in a boiling water bath for 10 min. After cooling at room temperature, SDS‐PAGE was injected into the sample hole according to 5∼10 μl in each hole. The parameters of the electrophoresis instrument were set to 80 V, and 30 min was used to concentrate the gel; then set to 120 V, and 1 h was used to separate the gel. The membrane was transferred to a constant current, rinsed for 5 min, and blocked with 5% skimmed milk powder at room temperature for 2 h. Added primary antibodies include GSNOR (1:800, Proteintech Cat# 11051‐1‐AP, RRID:AB_593422), TET1 (1:2000, Bioworld, BZ12161), and β‐actin (1:1000, Zs‐BIO, TA‐09). After incubation overnight at 4°C, wash with PBST. Blots were subsequently incubated with horseradish peroxidase‐conjugated secondary antibody (goat anti‐rabbit IgG: 1:5000, Zs‐BIO, ZB‐2301) for 2 h at room temperature, and then washed with PBST. The target protein bands of immune response were visualized using the ECL Kit (Thermo, USA), then use Image J software (Media Cybernetics, USA) to analyze the results of protein bands, and calculate the relative expression.

### 2.7 Statistical analysis

SPSS 22.0 statistical software was used for data analysis, and x ± s was used to describe the continuity data of the normal distribution. Repeated‐measures analysis of variance was used to analyze the data from the MWM learning task, with day, group, or age as the independent variable. The memory percentage of the distance from the MWM test and the target mRNA or protein expression levels were evaluated by one‐way ANOVA with age or treatment as independent variables, and comparison between groups used the LSD test. The correlation between GSNOR content and behavioral data was analyzed by Pearson correlation analysis. *p* < 0.05 is considered statistically significant.

## 3 RESULTS

### 3.1 Performance in the Morris water maze

#### 3.1.1 Learning phase

**FIGURE 2:**
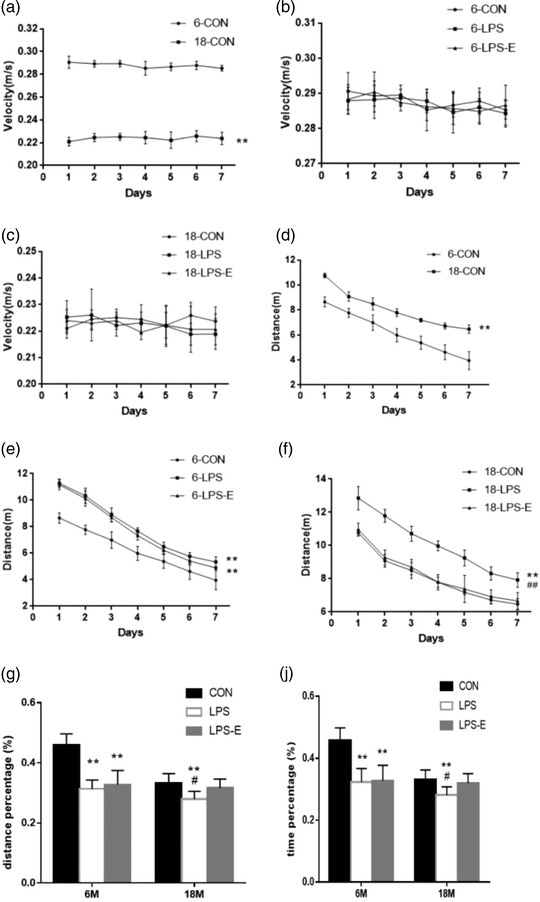
Performance in the Morris water maze (MWM) test. Average velocity (a,c,e), and distance (b,d,f) during the learning phase; percent distance swam and percent time swam (g,j) during the memory phase. Age and treatment had a significant effect on learning and memory performance in the MWM. Data are expressed as the mean ± SEM (*n* = 10 mice/group). ^*^Significant differences compared with 6‐ or 18‐month‐old CON mice (^*^ *p* < .05, ^**^ *p* < .01); ^#^Significant differences compared with 6‐ or 18‐month‐old LPS‐E mice (^#^ *p* < .05, ^##^ *p* < .01). 6, 6‐month‐old; 18, 18‐month‐old; CON, control group; LPS, lipopolysaccharide treatment group; LPS‐E, LPS plus enriched environment treatment group

When all mice were combined, the rm‐ANOVA results show that as the number of training days increased, the swimming distance (*F*_[6,114]_ = 1183.986, *p* < .01) gradually decreases; the average speed *(F*_[6, 114]_ = 0.113, *p* = .485) decreases with time, but it is not significant. The 18 M mice swam for a significantly longer distance (*F*_[1,58]_ = 39.252, *p* < .01) than the 6 M mice, the LPS mice swam for a significantly longer distance (*F*_[1,38]_ = 33.147, *p* < .01) than the CON mice, the LPS‐E mice swam for a significantly shorter distance (*F*_[1,38]_ = 33.147, *p* < .01) than the LPS mice. Moreover, the 18 M‐CON mice swam for a significantly longer distance (*F*_[1,18]_ = 475.814, *p* < .01) and had a slower swimming velocity (*F*_(1,18)_ = 803.290, *p* < .01) than the 6 M‐CON mice (Figure 2a,d). LPS and EE had no significant effect on the swimming speed of 6 and 18 M mice (Figure 2b,c). Therefore, swimming distance is more suitable as an indicator of learning ability. There were significant differences in the swimming distance among the treatment groups for both the 6 M mice (*F*_(2,27)_ = 299.718, *p* < .01; Figure 2e) and the 18 M mice (*F*_(2,27)_ = 190.557, *p* < .01; Figure 2f). In the two age groups, the swimming distance of the LPS group mice was significantly longer than that of the CON group mice (*ps* < .01). In addition, mice in the LPS‐E group swam a similar distance as the 6 M LPS mice (*p* = .159) and the 18 M CON mice (*p* = .150). The effect of the interaction of group × day was not significant in the learning phase (*ps* > .05).

#### 3.1.2 Memory phase

When all mice were combined, the percentage of swimming distance (*t* = 3.716, *p* < .01) and the percentage of swimming time (*t* = 3.829, *p* < .01) in the target quadrant of 18 M mice was significantly lower than that of 6 M mice, There were significant differences in the percentage of swimming distance (*F*_(2,57)_ = 19.955, *p* < .01) and the percentage of swimming time (*F*_(2,57)_ = 15.880, *p* < .01) among different treatment groups. The percentage of swimming distance (*t* = 8.173, *p* < .01) and the percentage of swimming time (*t* = 6.425, *p* < .01) in the target quadrant of 18 M CON mice was significantly lower than that of 6 M CON mice. There were significant differences in the percentage of swimming distance and the percentage of swimming time between the treatment groups for both the 6‐month‐old mice (*F_(_*_2,27)_ = 43.117, *p* < .01; *F_(_*_2,27)_ = 29.314, *p* < 0.01) and the 18‐month‐old mice (*F*_[2,27]_ = 8.323, *p* < .01; *F*_[2,27]_ = 7.806, *p* < .05).In the two age groups, the swimming distance percentage of the LPS group mice was significantly lower than that of the CON group mice (*ps* < .01). Post‐hoc analysis revealed that mice in the LPS‐E groups had similar percent distance swam and percent time swam as the 6‐month‐old mice in the LPS group (*p* = .432; *p* = .850) and the 18‐month‐old mice in the CON group (*p* = .241; *p* = .386; Figure 2g,j).

### 3.2 The mRNA levels of *Tet1* and *Gsnor* in the hippocampus

When all mice were combined, the 18 M group had significantly lower mRNA levels of TET1 (*t* = 10.205, *p* < .001) and GSNOR (*t =* 9.550, *p* < .001) than the 6 M groups, there were significant differences in the mRNA levels of GSNOR (*F*_(2,33)_ = 3.594, *p* < .05) among different treatment groups.

#### 3.2.1 Age effects

The 18 M CON group had significantly lower mRNA levels of TET1 (*t* = 4.306, *p* = .002) and GSNOR (*t =* 23.237, *p*< .001) than the 6 M CON group.

#### 3.2.2 Treatment effects

**FIGURE 3:**
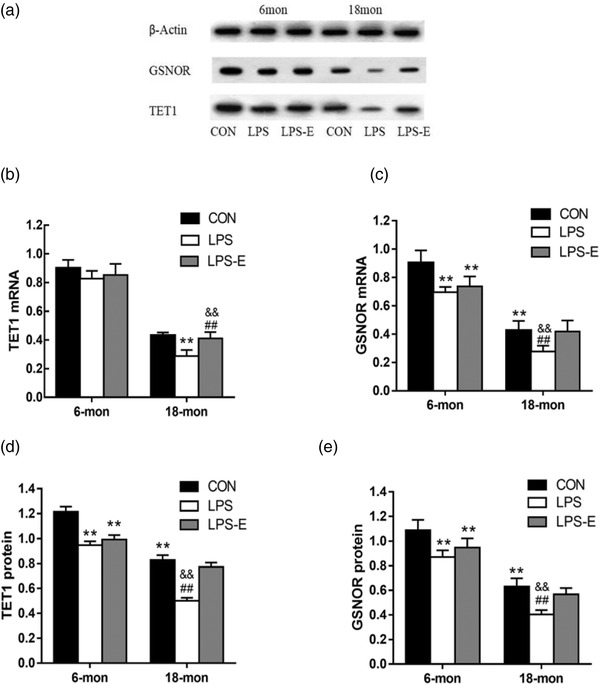
Relative mRNA and protein levels of *Tet1* and *Gsnor* in the hippocampus. (a) The protein bands after Western blotting. (b,c) Differences in *Tet1* and *Gsnor* mRNA levels in the different groups. (d,e) Differences in TET1 and GSNOR levels in the different groups. ^*^Significant differences compared with 6‐month‐old CON mice (^**^ *p* < .01); ^&^Significant differences compared with 18‐month‐old CON mice (^&&^ *p* < .01); ^#^Significant differences compared with 18‐month‐old LPS‐E mice (^#^ *p* < .05, ^##^ *p* < .01) (*n* = 6 mice/group). CON, control group; LPS, lipopolysaccharide treatment group; LPS‐E, LPS plus enriched environment treatment group

In 6 M mice, only GSNOR mRNA (*F*_[2,15]_ = 36.421, *p* < .001) levels differed among the groups; the expression of LPS‐E and LPS group was significantly lower than that of the CON group (*ps* < .001), and the expression of LPS‐e group was slightly higher than that of LPS group (*p* = .053).In 18 M mice, the levels of both mRNAs (*F*_[2,15]_ = 15.411, *p* < .001 for TET1; *F*_[2,15]_ = 14.465, *p* < .001 for GSNOR) differed significantly among the groups. Compared with the LPS‐E group and the CON group, the LPS treatment group had lower mRNA levels (*ps* < .001) of the two genes; no difference was found between the first two groups (*ps* >.05; Figure 3b,c).

### 3.3 The protein levels of TET1 and GSNOR in the hippocampus

When all mice were combined, the 18 M group had significantly lower levels of TET1 (*t* = 9.428, *p* < .001) and GSNOR (*t =* 8.164, *p* < .001) than the 6 M groups, there were significant differences in the levels of TET1 (*F*_(2,33)_ = 5.114, *p* < .05) and GSNOR (*F*_(2,33)_ = 6.395, *p* < .05) among different treatment groups.

#### 3.3.1 Age effects

The 18 M CON group had significantly lower protein levels of TET1 (*t* = 4.306, *p* = .002) and GSNOR (*t* = 23.237, *p* < .001) than the 6 M CON group.

#### 3.3.2 Treatment effects

Irrespective of age, different treatments significantly affected the levels of hippocampal TET1 (*F*_[2,15]_ = 322.940, *p* < .001 in 6 M mice; *F*_[2,15]_ = 24.474, *p* < .001 in 18 M mice) and GSNOR (*F*_[2,15]_ = 392.495, *p* < .001 in 6 M; *F*_[2,15]_ = 26.327, *p* < .001 in 18 M mice). Post‐hoc analysis showed that the levels of TET1 and GSNOR in the 6 M LPS and LPS‐E groups were significantly lower than those in the same‐age control group (*ps* < .001); there were marginally higher levels in the LPS‐E group than in the LPS group (*p* = .153 for TET1; *p* = .098 for GSNOR). In the 18 M, the two protein levels in the LPS treatment group were lower (*p*s < .001) than the LPS‐E group and CON group; there was no difference between the latter two groups (*ps <* .01, Figure 3d,e).

### 3.4 Correlations between performance in the MWM and the protein or mRNA levels of TET1 and GSNOR

The correlations between the hippocampal levels of TET1 and GSNOR and learning and memory in the MWM task were related to age and treatment, so each group was analyzed separately (Table 2).

**TABLE 2:** Correlations between performance in the MWM and TET1/GSNOR in the hippocampus

Performances	Ages	Groups	*Tet1* mRNA	*Gsnor* mRNA	TET1 protein	GSNOR protein
Swimming	6‐months	CON	−0.129(0.454)	−0.102(0.555)	−0.754(0.083)	−0.663(0.151)
distance		LPS	−0.627(0.183)	−0.522(0.288)	−0.625(0.184)	−0.019(0.971)
		LPS‐E	−0.677(0.140)	−0.264(0.613)	−0.335(0.517)	−0.660(0.154)
	18‐months	CON	−0.921(0.009)^**^	−0.910(0.012)*	−0.959(0.002)^**^	−0.836(0.038)*
		LPS	−0.885(0.019)*	−0.851(0.032)*	−0.957(0.003)^**^	−0.822(0.045)*
		LPS‐E	−0.860(0.028)*	−0.929(0.007)^**^	−0.847(0.033)*	−0.866(0.026)*
Distance	6‐months	CON	0.319(0.058)	0.241(0.156)	0.080(0.881)	0.479(0.336)
percentage		LPS	0.758(0.081)	0.790(0.062)	0.387(0.448)	0.251(0.632)
		LPS‐E	0.772(0.072)	0.033(0.950)	0.204(0.698)	0.695(0.125)
	18‐months	CON	0.786(0.064)	0.749(0.086)	0.718(0.108)	0.926(0.008)^**^
		LPS	0.867(0.025)*	0.832(0.040)*	0.774(0.071)	0.670(0.145)
		LPS‐E	0.706(0.117)	0.613(0.196)	0.760(0.080)	0.843(0.035)*

* Denotes significant correlation coefficients (^*^ *p* < .05; ^**^ *p <* .01).

Irrespective of the group, during the learning period the mRNA and protein levels of TET1 and GSNOR in the hippocampus of the 6 M mice were not significantly correlated with the mean swimming distance in the MWM (*p*s > .05). In contrast, the levels of mRNA and protein of TET1 and GSNOR in the hippocampus of the 18 M mice in these groups were negatively correlated with the mean swimming distance (*P*s < .05).

During the memory period, there was no significant correlation between TET1 and GSNOR mRNA and protein levels in the hippocampus of the 6‐month‐old mice and the percentage of distance swam in the target quadrant (*p*s > .05). In 18‐month‐old mice, hippocampal GSNOR protein in the control and LPS‐E groups and TET1/GSNOR mRNA in the LPS group were positively correlated with the percentage of distance in the target quadrant (*p*s < .05).

## 4 DISCUSSION

Pregnancy is a special and sensitive period, during which females are vulnerable to infections from bacteria and viruses (Li et al., 2016). LPS is a powerful endotoxin commonly used to simulate bacterial infections in laboratory animals, which can provide continuous inflammatory stimulation (Li et al., 2016). Pregnant animals are more sensitive to LPS, and LPS triggers maternal immune activation, leading to abnormal gene expression in key brain regions of the mother and fetus (Oskvig et al., 2012). Our previous studies suggested that inflammatory insults during pregnancy could be an important risk factor for the development of AACD (Li, Wang et al., 2016). The results of this experiment showed that exposure to LPS during pregnancy can accelerate cognitive impairment in elderly mice, and EE can alleviate this effect. Therefore, avoiding gestational infection is of great significance for protecting cognitive ability in old age, and long‐term environmental enrichment could mitigate the cognitive impairment induced by gestational inflammation. Moreover, we also found that the decreased expression of GNSOR and TET1 in the hippocampus was associated with impaired cognitive function in different treatment groups. Our results provide new insights into the mechanisms involved in the accelerated cognitive decline that results from gestational inflammation.

### 4.1 EEs alleviate the accelerated spatial cognition impairment in aged females caused by gestational inflammation

In both humans and rodents, cognitive functions such as spatial learning and memory gradually decline during aging (Yassa et al., 2011). The current results indicating that learning and memory abilities gradually decrease with age are in line with many previous results (Cao et al., 2013; Chen et al., 2011; Duan et al., 2020; Ennaceur et al., 2008; Khan et al., 2021; Wu et al., 2020). In fact, our previous studies showed that CD‐1 mice began to experience cognitive impairments at 12 months of age (Li, Cao et al., 2016; Wu et al., 2019).

LPS, the cell wall component of Gram‐negative bacteria, can activate the immune system and induce the expression of proinflammatory cytokines (Dantzer, 2004; Li et al., 2016). These cytokines can affect the normal function of the brain through specific signaling pathways and accelerate brain aging (Akbarian et al., 2013; Patterson, 2015; Zhuang et al., 2020). In the present study, mice exposed to inflammation during pregnancy performed significantly worse than normal mice on the MWM test at 6 months and 18 months. The current results suggested that gestational inflammation can accelerate age‐associated cognitive impairment in both learning and memory. Moreover, the LPS and LPS‐E mice had worse learning and memory performance than the CON mice at both ages. Nevertheless, the LPS‐E mice had similar learning and memory performance as the 6 M LPS mice and had significantly better learning and memory performance than the 18 M LPS mice. Therefore, our evidence suggested that living in EE can significantly reduce the accelerated spatial cognitive impairment in both learning and memory resulting from gestational inflammation of elderly LPS mice, but it takes enough time to achieve this effect (at least more than 3 months).

### 4.2 EEs slow the accelerated decrease of hippocampal TET1/GSNOR in aged mice with gestational inflammation

Oxidative stress is a condition in which endogenously or exogenously produced pro‐oxidant species—for example, reactive oxygen and nitrogen species (ROS and RNS, respectively)—overwhelm the antioxidant defense during basal conditions (Sies et al., 2017; Sies & Cadenas, 1985). Dysfunction of the body's response to oxidative stress results in the accumulation of damaged biomolecules and cellular structures (Singh et al., 2021), which are hallmarks of cell senescence. In this scenario, mitochondria play a fundamental role as they are the main intracellular source of oxygen free radicals along with being one of the main targets of ROS and RNS (Balaban et al., 2005).

Mitochondrial aging free radical theory is that aging is the result of mitochondrial damage accumulation and that mitochondrial dysfunction is an important process for a variety of age‐related disease pathophysiologies (Montagna et al., 2020, 2014). Mitochondria have a large number of protein cysteine residues. These residues can be oxidized by nitric oxide (NO) to form S‐nitrosothiols (SNOs) in a process called S‐nitrosylation. The oxidized form of cysteine is reversible, which makes cysteine act as a redox sensor or redox switch (Hess & Stamler, 2012; Rizza & Cardaci et al., 2018b). This oxidative modification changes the activity of related proteins and regulates mitochondrial function (Ischiropoulos, 2009). Excessive S‐nitrosylation of related proteins will directly affect mitochondrial dynamic activity, reduce the efficiency of aerobic metabolism, inhibit mitochondrial autophagy, and destroy mitochondrial homeostasis (Liu et al., 2001b). S‐nitrosoglutathione (GSNO), a low‐molecular‐weight SNO, can be reduced back to a sulfhydryl state by denitrosylation reactions and then affect the concentration of protein SNOs (2014). GSNOR is a highly specific denitrase for GSNO; it controls the intracellular levels of GSNO and protein SNOs and protects the body from nitrosative stress. GSNOR expression has been found to reduce the primary aging cells that are accumulated during the rodent and human aging process (Montagna et al., 2014). Recently, some have reported that GSNOR, by means of regulating the S‐nitrosylation state of proteins involved in mitochondrial dynamics and mitophagy, sustains the removal of irreversibly damaged mitochondria and delays cell senescence (Rizza & Filomeni, 2018). Mice with Gsnor defects (Gsnor ^‐/−^) have a greatly increased degree of brain protein S‐nitrosylation and show some characteristics of accelerated aging such as decreased muscle mass, neuromuscular dysfunction, and positive α‐synaptic nuclear protein accumulation in the cerebral cortex (Montagna et al., 2014; Rizza & Filomeni, 2018). Therefore, decreased expression of GSNOR may be an important indicator of aging.

The *Gsnor* promoter has a CpG island, which is an epigenetic predictive marker of cytosine methylation (Rizza & Cardaci et al., 2018b). The expression of *Gsnor* is regulated by TET1. TET1 catalyzes the demethylation of the methylated cytosine of the *Gsnor* promoters. Oxidation of 5‐methylcytosine (5meC) to 5‐hydroxymethylcytosine (5hmeC) is the first reaction catalyzed by TET1 to reduce methylated cytosine and then promote *Gsnor* expression. 5hmeC is a molecular marker of active cytosine demethylation. As age increases, an increase in 5mC and a decrease in 5hmC indicate that the TET1 and GSNOR axis may play an important role in aging (Yassa et al., 2011). TET1 can be inhibited by succinic and fumaric acids, which are metabolites produced by the tricarboxylic acid cycle (Ferrer et al., 2020; Laukka et al., 2016; Xiao et al., 2012). Excessive S‐nitrosylation caused by GSNOR deficiency may lead to the accumulation of fumaric acid and further inhibit TET1 activity (Zeng et al., 2015), which suggests that reduced activity of TET1 can lead to methylation of the *Gsnor* promoter and further reduce GSNOR expression. Because senescence is associated with an increase in 5mC and a decrease in 5hmC in the CpG island within the promoter (Rizza & Cardaci et al., 2018b), we hypothesized that TET1 and GSNOR are closely related in the senescence process.

Some studies have shown an age‐related decrease in TET1 and 5hmeC in peripheral blood mononuclear cells and T cells (Petursdottir et al., 2006; Truong et al., 2015; Valentini et al., 2016). These results are consistent with ours, which showed that the mRNA and protein levels of hippocampal TET1 and GSNOR in the aged (18 M) CON mice were significantly reduced compared with those in the young (6 M) CON mice. This suggested that age significantly affects the expression of TET1 and GSNOR. Moreover, gestational inflammation exerted age‐dependent effects on hippocampal *Tet1* and *Gsnor* expression. Specifically, at 6 months of age LPS mice had significantly decreased levels of *Gsnor* mRNA and protein and *Tet1* protein than the CON mice, while at 18 months of age LPS mice had significantly decreased levels of both *Tet1* and *Gsnor* mRNA and protein than the CON mice. These results suggested that LPS exposure during pregnancy appears to decrease *Tet1* and *Gsnor* expression in the hippocampus of mice, especially aged mice. Consequently, decreased expression of *Tet1* and *Gsnor* would increase S‐nitrosylation of mitochondrial protein, which would lead to mitochondrial dynamic disorders and result in abnormal mitochondria morphology and dysfunction as well as inhibit mitochondrial autophagy (Rizza & Cardaci et al., 2018a, 2018b).

Interestingly, compared to LPS mice, LPS‐E mice had similar levels of mRNA and protein of *Gsnor* and *Tet1* at 6 months of age and increased levels at 18 months of age. When compared with the CON mice, LPS‐E mice had increased levels of mRNA and protein of *Gsnor* and *Tet1* at 6 months of age and similar levels at 18 months of age. These findings indicated that only long‐term EE exposure can drive the otherwise accelerated decrease in hippocampal expression of *Tet1* and *Gsnor* caused by gestational inflammation to near normal levels, especially in the aged hippocampus. Therefore, EEs need to be provided over a long time period to reverse the future consequences of gestational inflammation.

The mechanisms of how EEs increase the expression of *Tet1* and *Gsnor* are unknown and complex at the very least. The effects of how EEs delay brain aging have been related to many factors. All of these factors may contribute to the increased expression of *Tet1* and *Gsnor* in the mice living in EEs. For example, EEs can increase brain‐derived neurotrophic factors, promote the growth and maturation of neurons, and increase resistance to brain insult (Shlevkov et al., 2016). EEs can also alter the expression of important genes involved in brain aging through modifications in acetylation or DNA methylation patterns (Ikegami & Narita, 2012; Zhuang et al., 2020). In addition, EEs induce the production of proteins that suppress oxyradical production (Fernández CI, 2004). EEs include adequate conditions and opportunities for getting food and exercise. Studies have shown that going for long periods with no breakfast can significantly inhibit the mRNA of hippocampal memory‐related genes such as *Tet1* (Okauchi et al., 2020) and that a healthy lifestyle from activities such as long‐term regular exercise can increase hippocampal *Tet1* mRNA in rats (Sølvsten et al., 2018). These studies indirectly support the promotion of hippocampal *Tet1* and *Gsnor* expression by EEs.

### 4.3 Hippocampal reduction of TET1 and GSNOR are associated with impaired cognition

Mitochondria are critical for neuronal energy metabolism, calcium regulation, myelination, and neuronal and glial apoptosis (Princz et al., 2018; Zhuang et al., 2020). Therefore, mitochondrial decay may be central to neuronal damage and cognitive decline (Cheng et al., 2018; Lai et al., 2017). In the aging process of rodents and humans, Gsnor expression is reduced. This reduction is associated with mitochondrial stress damage, which is characterized by increased S‐nitrosylation of mitochondrial protein (Larrick & Mendelsohn, 2019). *Tet1* knockout mice showed a reduction in multiple neuron activity‐regulating genes and obvious damage to learning and memory (Rudenko et al., 2013; Yang et al., 2017) . Therefore, TET1 and GSNOR may play an important role in the age‐related decline in learning and memory.

As expected, the current experiment found that there were close connections between the age‐related decline in learning and memory and TET1 and GSNOR in the aged hippocampus. In senescent (18‐month‐old) mice, these correlations were treatment‐dependent. The aged mice in the CON group showed a negative correlation between the levels of *Tet1* and *Gsnor* mRNA and protein with the learning‐phase distance swam, and a positive correlation between the levels of *Tet1* and *Gsnor* mRNA and protein with the memory‐phase percent distance swam. Moreover, the LPS and LPS‐E mice showed similar correlations in the learning‐phase as the CON mice. However, there was only a positive correlation between the mRNA levels of *Tet1* and *Gsnor* and the memory‐phase percent distance swam in LPS mice and between the protein‐level of *Gsnor* and the percent memory‐phase distance swam in LPS‐E mice. Therefore, the impaired cognitive performance caused by exposure to inflammation during pregnancy may be related to decreased *Tet1* and *Gsnor* transcription, which then leads to decreased translation in the aged animals. This decreased translation can be modulated by gestational inflammation or subsequent EE exposure.

That the long‐term EE alleviated the accelerated cognitive decline may be attributable to increased *Gsnor* translation. Of note, in young mice (6 months old), there was no significant correlation between the learning‐phase distance swam or the memory‐phase percent distance swam and the protein and mRNA levels of *Tet1* and *Gsnor*, regardless of treatment. We speculated that the mitochondrial damage with gestational inflammation in 6‐month‐old mice was not enough to cause changes in learning and memory due to unknown compensatory mechanisms in the brain. The impaired spatial learning and memory abilities in young mice (6 months old) caused by gestational inflammation may be related to complex mechanisms such as changes in hippocampal mitochondrial biogenesis and dynamics as well as synaptic plasticity (Li, Cao et al., 2016; Zhuang et al., 2020).

## 5 SUMMARY

The expression of hippocampal *Tet1* and *Gsnor* in mice showed an age‐related decline. Exposure to LPS during pregnancy accelerated the down‐regulation of hippocampal *Tet1* and *Gsnor* in aged mice, while a long‐term EE had a protective effect on this damaging outcome. Therefore, providing long‐term positive life events such as EE or avoiding negative life events such as pregnancy infection has important clinical significance for the prevention and treatment of age‐related cognitive impairment. Our study had some limitations. First, there were fewer groups of aged mice. Second, this study only determined the expression of *Tet1* and *Gsnor* but did not further explore the associated signaling pathways. However, despite these limitations, we provide new insights into the mechanisms of cognitive impairment caused by gestational infection and the protective effects caused by almost lifelong EE exposure. Future research is needed for further clarification.

## AUTHOR CONTRIBUTIONS

ZYX conceived and designed the study, conducted literature search and data collection, analyzed data, and wrote manuscripts; WQY, WYT, and ZLP assisted in data collection and preliminary analysis; CL and CGH reviewed the manuscript. All the authors read and approved the content of the manuscript.

## CONFLICT OF INTEREST

The authors declare that the research was conducted in the absence of any commercial or financial relationships that could be construed as a potential conflict of interest.

### PEER REVIEW

The peer review history for this article is available at https://publons.com/publon/10.1002/brb3.2817.

## Data Availability

The data that support the findings of this study are available from the corresponding author upon reasonable request.
